# PDSTD - The Portsmouth Dynamic Spontaneous Tears Database

**DOI:** 10.3758/s13428-021-01752-w

**Published:** 2021-12-16

**Authors:** Dennis Küster, Marc Baker, Eva G. Krumhuber

**Affiliations:** 1 Department of Mathematics and Computer Science, University of Bremen, Enrique-Schmidt Str. 5, 28359 Bremen, Germany; 2 Department of Psychology, University of Portsmouth, Portsmouth, UK; 3 Department of Experimental Psychology, University College London, London, UK

**Keywords:** facial expression, emotion, dynamic, database, sadness, crying

## Abstract

The vast majority of research on human emotional tears has relied on posed and static stimulus materials. In this paper, we introduce the Portsmouth Dynamic Spontaneous Tears Database (PDSTD), a free resource comprising video recordings of 24 female encoders depicting a balanced representation of sadness stimuli with and without tears. Encoders watched a neutral film and a self-selected sad film and reported their emotional experience for 9 emotions. Extending this initial validation, we obtained norming data from an independent sample of naïve observers (*N* = 91, 45 females) who watched videos of the encoders during three time phases (neutral, pre-sadness, sadness), yielding a total of 72 validated recordings. Observers rated the expressions during each phase on 7 discrete emotions, negative and positive valence, arousal, and genuineness. All data were analyzed by means of general linear mixed modelling (GLMM) to account for sources of random variance. Our results confirm the successful elicitation of sadness, and demonstrate the presence of a *tear effect*, i.e., a substantial increase in perceived sadness for spontaneous dynamic weeping. To our knowledge, the PDSTD is the first database of spontaneously elicited dynamic tears and sadness that is openly available to researchers. The stimuli can be accessed free of charge via OSF from https://osf.io/uyjeg/?view_only=24474ec8d75949ccb9a8243651db0abf.

## Introduction

While Darwin regarded emotional weeping merely as an incidental response fulfilling non-emotional functions such as the lubrication and protection of the eyes (Darwin, 1872; Vingerhoets, 2013), there is little doubt today that emotional tears serve important social signalling functions (Gračanin et al., 2021). Through the act of crying, we intuitively reach out to others, thereby eliciting prosocial responses from onlookers (Hendriks et al., 2008; Zickfeld et al., 2020). Tears may act as a social glue that heightens both the perceived helplessness of the weeper and feelings of connectedness from observers (Vingerhoets et al., 2016). The *riddle of tears* (Vingerhoets & Bylsma, 2016), in particular the interplay between tears and facial expressions, has thus increasingly piqued the interest of researchers studying the social, cultural, and evolutionary role of emotional tears in human communication (Gračanin et al., 2018; Hasson, 2009; Sharman et al., 2020; Zickfeld et al., 2020). The present work aims to contribute to our understanding of tears as a socio-emotional signal by presenting the first validated and openly available database of spontaneously elicited tears and dynamic sadness expressions: the Portsmouth Dynamic Spontaneous Tears Database (PDSTD).

### Spontaneous Tears

In recent years, there has been an increasing demand for spontaneous expression stimuli in the behavioral sciences (Krumhuber et al., 2017; Küster et al., 2020; Sato et al., 2019). While spontaneous expressions are more difficult to experimentally control, they provide numerous advantages in terms of ecological validity. Besides capturing the realistic nature of everyday behavior (Zeng et al., 2009), spontaneous displays allow for more subtle and non-prototypical forms of expression. Unfortunately, however, most studies to date have focused on prototypical and posed displays of tears (Krivan & Thomas, 2020).

By digitally adding or removing tears from still images (Küster, 2015; Takahashi et al., 2015) this approach has provided important insights as to how emotional tears impact human observer judgments (Hendriks & Vingerhoets, 2006; Vingerhoets et al., 2016; Zickfeld & Schubert, 2018). For example, studies using posed and digitally manipulated tears have demonstrated the *tear effect*, a substantial increase in ratings of sadness for tearful relative to tear-free faces (Provine et al., 2009). However, since none of the subjects depicted in the stimulus materials (hereafter called “encoders”) actually cried in any of the images, it is questionable to what extent the results are applicable to real interpersonal and emotional communication. Also, artificially added tears may lack genuineness (Krivan & Thomas, 2020) and realism (Küster, 2018) in the sense that digitally manipulated tears exaggerate (e.g., size of tears) or miss relevant cues (e.g., eye redness). Studies using digitally removed tears offer somewhat greater ecological validity (Krivan & Thomas, 2020; Picó et al., 2020). However, with images often being sourced from the Internet (e.g., Flickr; Provine et al., 2009; Takahashi et al., 2015), several types of selection biases may arise. These include (self-)selecting images for upload, the impact of online engagement on search engines, and subsequent researcher selection. In consequence, it is unknown whether encoders truly felt sadness and/or experienced their tears as authentic signals of an underlying affective state (Krivan & Thomas, 2020). It is therefore unclear to what extent the effects of digitally posed tears on human observers may generalize to more naturalistic contexts.

So far, there exists only one set of spontaneous crying stimuli in which encoders responded to an emotion-eliciting situation. Originally recorded at an exhibition by Marina Abramović at the Museum of Modern Art (MOMA) in 2010 (van de Ven et al., 2017), the set consists of images depicting visitors who spontaneously cried during an interaction with the artist. While these stimuli are being employed in a growing number of studies (Gračanin et al., 2018; Picó et al., 2020; Riem et al., 2017; van de Ven et al., 2017; Vingerhoets et al., 2016; Zickfeld & Schubert, 2018), it could be argued that the criers were in a rather unusual ‘on stage’ situation that was highly public. Hence, it is unclear whether and to what extent these materials are comparable to tears shed in more private contexts. Also, due to the specific nature of the setting no self-report ratings were obtained from the encoders, which poses a limitation in terms of stimulus validation.

### Dynamic Expressions

Facial expressions are highly dynamic phenomena capable of conveying complex psychological states. The motion inherent in dynamic stimuli is crucial for social perception and improves coherence in identifying facial affect (Krumhuber et al., 2013; Krumhuber & Skora, 2016; Orlowska et al., 2018). To learn more about the determinants of crying (Vingerhoets, 2013; Vingerhoets & Bylsma, 2016), dynamic stimuli could provide rich information about the temporal context and behavioral antecedents of crying. For example, being able to observe criers over time, especially in the moments before the first appearance of their tears, may reveal a broad range of socio-emotional factors. A database containing tearful expressions as stimuli may thus contribute to perception studies, as well as to research on the response dynamics that are already encoded in these materials.

Interestingly, only a few studies to date have incorporated dynamic materials, either as part of laboratory research on weeping (Gračanin et al., 2015; Hendriks et al., 2007; Ioannou et al., 2016; Rottenberg et al., 2003; Sharman et al., 2020) or by means of videotaped case studies (Capps et al., 2013). Some of this research has revealed important insights about the intraindividual functions of crying for weepers. As such, emotional tears are invoked as part of a complex interplay between the sympathetic and parasympathetic nervous system (Ioannou et al., 2016), where they may help to maintain a state of biological homeostasis (Sharman et al., 2020). However, to the best of our knowledge, none of these studies have made their stimulus materials available to the public.

### Stimulus Validation

Current methods in crying research range from retrospective surveys (Bylsma et al., 2008), diary studies (Bylsma et al., 2011), and clinical observations (Capps et al., 2013) to experiments leveraging digitally manipulated tears (Krivan & Thomas, 2020) and examining the physiological correlates of weeping (Sharman et al., 2020). Due to the variety in approaches, the role of emotional tears in socio-emotional signalling is not well understood since a shared methodological basis has been missing so far. Here, spontaneously elicited dynamic expressions in the laboratory could facilitate more standardized materials for ecologically valid perception studies. This necessitates recording conditions that are technically controlled yet allow for spontaneous behavior to occur in a relatively unrestricted manner. Furthermore, participants need to provide informed extended consent to allow for their sensitive data to be shared between researchers. To date, such methodological choices in stimulus construction have been difficult to achieve, with either laboratory studies focusing on the encoding of tears/ sadness or decoding studies relying on photoshopped tears (Krivan & Thomas, 2020).

We think that an important first step towards more integrative research entails the validation of expressive stimuli by both encoders and decoders. At present, the crying images sourced from the MOMA exhibition (van de Ven et al., 2017) come the closest to a standardized set of spontaneous stimuli. However, while the MOMA picture set is lacking self-report data from encoders, videos obtained from encoding studies conversely still lack normative ratings from observers. This dearth of knowledge calls for validation studies that combine data from self-reports of subjective experience as well as observer judgments to provide a comprehensive resource for stimulus selection in crying research.

### Aims of the Present Research

The current work aims to address this gap in the literature by presenting and validating the first database of spontaneously elicited tears and sadness expressions: the PDSTD. It contains close-up video recordings of 24 female participants who watched self-selected sad film-clips as well as a standard neutral film. We instructed participants before the experiment to self-select a sad film and identify a sad scene that they either found very sad or in response to which they had previously cried. Each clip was presented only once, without participants being able to replay or skip any parts of the materials. Half of the recordings depict weepers, allowing for a balanced representation of sadness stimuli with and without tears as determined via infrared thermal imaging. We validated the facial expression stimuli in a two-fold manner by collecting self-report data from encoders as well as norming data from naïve observers.

For this, encoders reported their experience of nine emotions directly after watching each film-clip. If the study manipulation is successful, the idiosyncratic choice of sad film-clips should induce more negative feelings (i.e., sadness) compared to the neutral (control) film. We further explored the intrapersonal effects of tears by comparing weepers vs. non-weepers. While tearing is believed to fulfil a beneficial or otherwise ‘cathartic’ function for emotion regulation (Breuer & Freud, 1895/2009; Vingerhoets, 2013), the opposite could similarly hold true, with more negative emotions being experienced by weepers than non-weepers (Gračanin et al., 2015, but see Sharman et al., 2020).

In order to obtain norming data, we further collected ratings of the targets from naïve observers. These tapped into the core dimensions of valence and arousal (Russell, 1980), as well as discrete categories of the basic six emotions (Ekman, 1992). To create a measure of perceived ecological validity, observers also rated the emotion genuineness of the expressions. Given that all stimuli were dynamic in appearance, we explored whether observers are sensitive to the presence of weeping and its behavioral antecedents. To that end, we distinguished between two time phases during the sad film episode: shortly before the saddest moment/first tear (*pre-sadness*) and after the saddest moment/first tear (*sadness*). If early signs of weeping - prior to the actual presence of tears - are conveyed in the pre-sadness phase, observer ratings should be more negative compared to those of the neutral (control) baseline. In accordance with empirical work on the tear effect using static images, we further predicted a substantial increase in perceived negativity (i.e., negative valence, arousal, sadness, (Gračanin et al., 2021; Ito et al., 2019; Reed et al., 2015) of weepers (vs. non-weepers) during the sadness phase. This should be reflected in a significant interaction between weeping and time phase, such that weepers are perceived as most negative after they started crying.

In an effort to provide a full validation of the PDSTD, we aimed to control for random variance in rater identity, encoder identity, and the type of sad film-clip being selected by encoders. For example, individual raters may randomly differ from other observers in how they evaluate the stimulus materials. Hence, all our statistical analyses employed general linear mixed modelling (GLMM) which can be viewed as an extension of conventional statistical models (e.g., ANOVAs).

## Methods

### Stimulus development

#### Participants

As part of a larger study on thermal infrared imaging (Baker, 2019), female students (thereafter referred to as *encoders*) were recruited using social networking sites and through the University of Portsmouth recruitment database. We recruited female participants due to the substantially greater ease of eliciting weeping from female than male participants (e.g., Gračanin et al., 2015; Sharman et al., 2019). For the purpose of database construction, only encoders who provided an extended explicit written consent^1^ for the subsequent use of their video-recordings were included. This resulted in a total of 24 female encoders, primarily White (*n* = 22), ranging in age from 18-33 years (*M*_age_ = 21.50, *SD* = 3.22). Ethical approval was granted by the departmental ethics committee at the University of Portsmouth (reference number: SFEC 2018-011).

#### Procedure

**Fig. 1:**
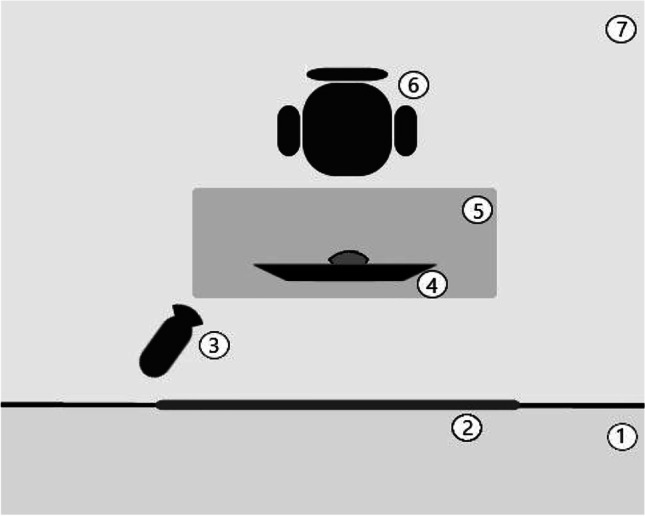
The Experimental Set-up Consisting of a (1) Control Room, (2) Mirrored Glass, (3) Thermal Imaging Camera, (4) Monitor with External Webcam, (5) Desk, (6) Chair, (7) Experimental Room

As weeping is notoriously difficult to generate under laboratory conditions (Vingerhoets & Bylsma, 2016), encoders were asked to bring a sad movie of their own choice and identify the scene they find most emotionally arousing (i.e., saddest). Table 1 details the respective films including the relevant scenes. In the study, encoders watched the self-selected sad film-clip (10-15 min) and a neutral film-clip about owls (approximately 5 min) alone in a sound-attenuated laboratory. Stimulus presentation was counter-balanced and was monitored from the adjoining control room that was invisible to encoders (cf. Fig. 1). We included a simple 10-min coloring task in-between both conditions to ensure that participants could return to an affectively and expressively neutral/baseline before watching the second video. Dynamic facial behavior was recorded using a Logitech C920 Pro HD webcam, with a video resolution of 1920 x 1080 and a frame rate of 30 fps.

**Table 1:** Encoders’ Self-Selected Sad Films and the Chosen Peak Arousal Scene

Sad film	Sad scene	No. of non-weepers	No. of weepers
Marley and Me	Marley dies	5	4
The Notebook	Noah and Allie die	2	1
Titanic	Elderly couple die/Jack dies	0	2
A Fault in our Stars	Augustus says his cancer has returned	1	1
My Sister’s Keeper	Kate reads a memory book	1	0
The Mist	David Drayton kills his family	1	0
Timbuktu	Kidane and his wife die	0	1
Harry Potter and the Half-Blood Prince	Dumbledore dies	0	1
Monsters Inc.	Sulley says goodbye to Boo	1	0
The Amazing Spiderman 2	Gwen Stacey dies	1	0
The Land Before Time	Littlefoot’s grandmother dies	0	1
Green Mile	John Coffey is taken to be executed	0	1

For Titanic, both scenes were indicated as peak moments and occurred within a short period of time.

After each film-clip, encoders rated their subjective experience for nine emotions (happiness, sadness, fear, anger, disgust, amusement, interest, boredom, and relaxation) on 10-point Likert scales (1 = *low*, 10 = *high*). For reasons of consistency, we transformed the scores into a 0-100 scale to allow for direct comparison with the observer ratings. All measures were presented on the same screen and in a fixed order, with unlimited response time. Heart rate, respiration rates and skin conductance were also recorded, but are not included in this paper.

**Fig. 2:**
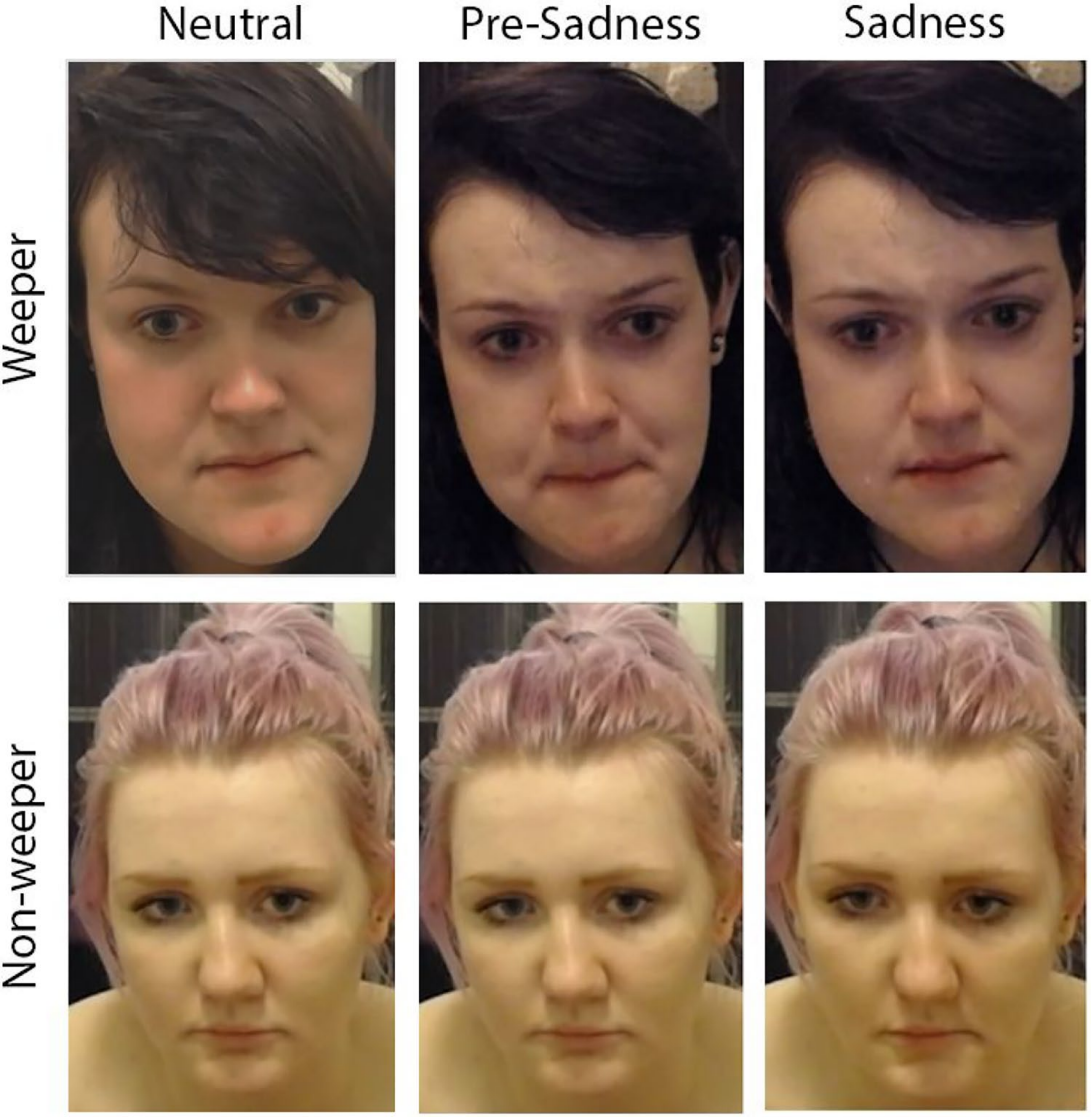
Example of Stimuli Depicting a Weeper and Non-Weeper During the Three Time Phases. *Note*. Stimuli are cropped here to allow for maximum visibility, with adjusted brightness levels of some images. The validation study used the full (non-cropped) videos

From the 24 encoders, half (12 females) wept spontaneously during the sad film-clip. Tear production was determined via infrared thermal imaging using a FLIR A655sc (Baker, 2019). For non-weepers we used the point in the film that had been indicated by each participant to be the saddest moment and treated it as the equivalent to weeping participants’ first tear. For each encoder, we extracted three 30 s segments from the recording: the end of the neutral film-clip (*neutral*), 30 s before the saddest moment / first tear (*pre-sad*), and from 10 s before to 20 s after the saddest moment / first tear (*sad*). The resulting 72 videos showed each encoder once during the neutral baseline (owl video), and twice during the sad film episode. Examples of the three phases are shown in Figure 2.

### Stimulus ratings

#### Participants

Ninety-five student raters were recruited face-to-face or via the departmental subject pool at University College London and received partial course credit. Responses from four participants were discarded as they failed an inbuilt attention-check (Oppenheimer et al., 2009). This resulted in a final sample of 91 raters (45 females, *M*_age_ = 21.88 years, *SD* = 4.06) from which the majority identified as White (82% White, 7% Asian, 11% Other/Mixed). All raters provided written informed consent prior to the study. Ethical approval was granted by the departmental ethics committee at University College London.

#### Procedure

Raters were tested individually on computers running Qualtrics, a web-based software (Provo, UT). Upon arrival, they were informed that the study aimed to explore how people perceive emotions in dynamic facial expressions. To avoid potential cueing effects due to the repeated presentation of the same encoder (Mishra & Srinivasan, 2017), raters only viewed one video per encoder, with an equal number of videos from phase 1, 2, and 3. The 24 videos to be evaluated (out of the 72) were presented one at a time in a randomized order.

For each video, raters indicated the degree of perceived negative and positive valence, arousal, and emotion genuineness on 100-point VAS scales (0 = *not at all*; 100 = *very much*). All four measures were presented on the same screen and in a fixed order. Arousal was defined as a dimension that goes from excited, wide-eyed, awake at the high end of the scale to relaxed, calm, sleepy at the low end. An expression was defined as “genuine” if the person appears to truly feel an emotion, in contrast to a “posed” expression which is simply put on the face while nothing much may be felt. Afterwards, they rated the extent (from 0 to 100%) to which each of the following emotions is reliably expressed in the face: anger, disgust, fear, happiness, sadness, surprise, and neutral. If they felt that more than one category applied, they could respond using multiple sliders to choose the exact confidence level for each emotion category. Ratings across the seven categories had to sum up to 100%. Response time was unlimited for all measures.

## Results

### Self-reports of encoders

To analyze encoders’ self-report ratings, a GLMM was built separately for each of the following emotion categories: sadness, happiness, anger, fear, disgust, interest, boredom, and relaxation. Encoder identity was treated as a nested variable within the sad film-clip, and a random slope was included at the level of film-clips to account for the idiographic nature of each film in inducing sadness. We entered condition (neutral vs. sadness), tearing (weeper vs. non-weeper) and the interaction between condition and tearing as fixed factors in the models. Table 2 displays the effect estimates for each of the models^2^. For reasons of brevity, we will only discuss the significant effects here.

**Table 2:** Estimates of Effects for Encoder Self-Ratings of Emotion

Effect	Sadness			Happiness			Fear			Anger			Disgust			Amusement			Relaxation			Interest			Boredom		
	Est.	*SE*	*p*	Est.	*SE*	*p*	Est.	*SE*	*p*	Est.	*SE*	*p*	Est.	*SE*	*p*	Est.	*SE*	*p*	Est.	*SE*	*p*	Est.	*SE*	*p*	Est.	*SE*	*p*
Fixed effects																											
Intercept (β_0j_)	15.75	5.16		54.67	6.08		0.92	1.70		0.00	3.12		7.33	5.71		32.42	6.70		57.42	5.95		43.42	7.39		24.00	6.42	
Sadness	**57.50**	7.29	**<.001**	**-24.17**	8.59	**.01**	4.97	4.44	.26	**11.59**	6.20	**.06**	-2.75	8.08	.73	-9.25	9.48	.33	-10.33	8.42	.22	**23.33**	10.45	**.03**	-14.75	9.09	.10
Weeper	7.25	7.29	.32	-11.25	8.59	.19	0.00	2.40	>.99	4.58	4.42	.30	11.17	8.08	.17	-0.08	9.48	.99	**-18.50**	8.42	**.03**	9.33	10.45	.37	-2.75	9.09	.76
Sadness * Weeper	1.17	10.31	.91	0.00	12.15	>.99	7.52	5.00	.13	-7.49	8.04	.35	-2.75	11.42	.81	-18.50	13.40	.17	-8.33	11.90	.48	**-30.75**	14.78	**.04**	-6.50	12.85	.61
Random Effects																											
Sad Film (*U*1j)	0.00	0.00		0.00	0.00		204.17	76.43		241.08	113.55		0.00	0.00		0.00	0.00		0.00	0.00		0.00	0.00		0.00	0.00	
Residual (***e***_ij_)	319.11	65.14		443.14	90.46		34.50	9.06		117.14	30.73		391.43	79.90		538.92	110.01		424.94	86.74		655.59	133.82		495.26	101.10	
-2*loglikelihood	412.96			428.73			345.78			388.50			422.77			438.12			423.71			447.52			434.06		

Estimates printed in bold indicate statistically significant effects at *p* < .05, with the neutral phase and non-weepers acting as reference groups (i.e., the intercept). *Sadness* represents the effect of the sad phase for non-weepers compared to the neutral phase. *Weeper* represents the effect of being a weeper during the neutral phase, and *Sadness* * *Weeper* represents the effect of weepers compared to non-weepers in the sad phase. *Sad Film* refers to the random effect of watching a specific sad film.

**Fig. 3:**
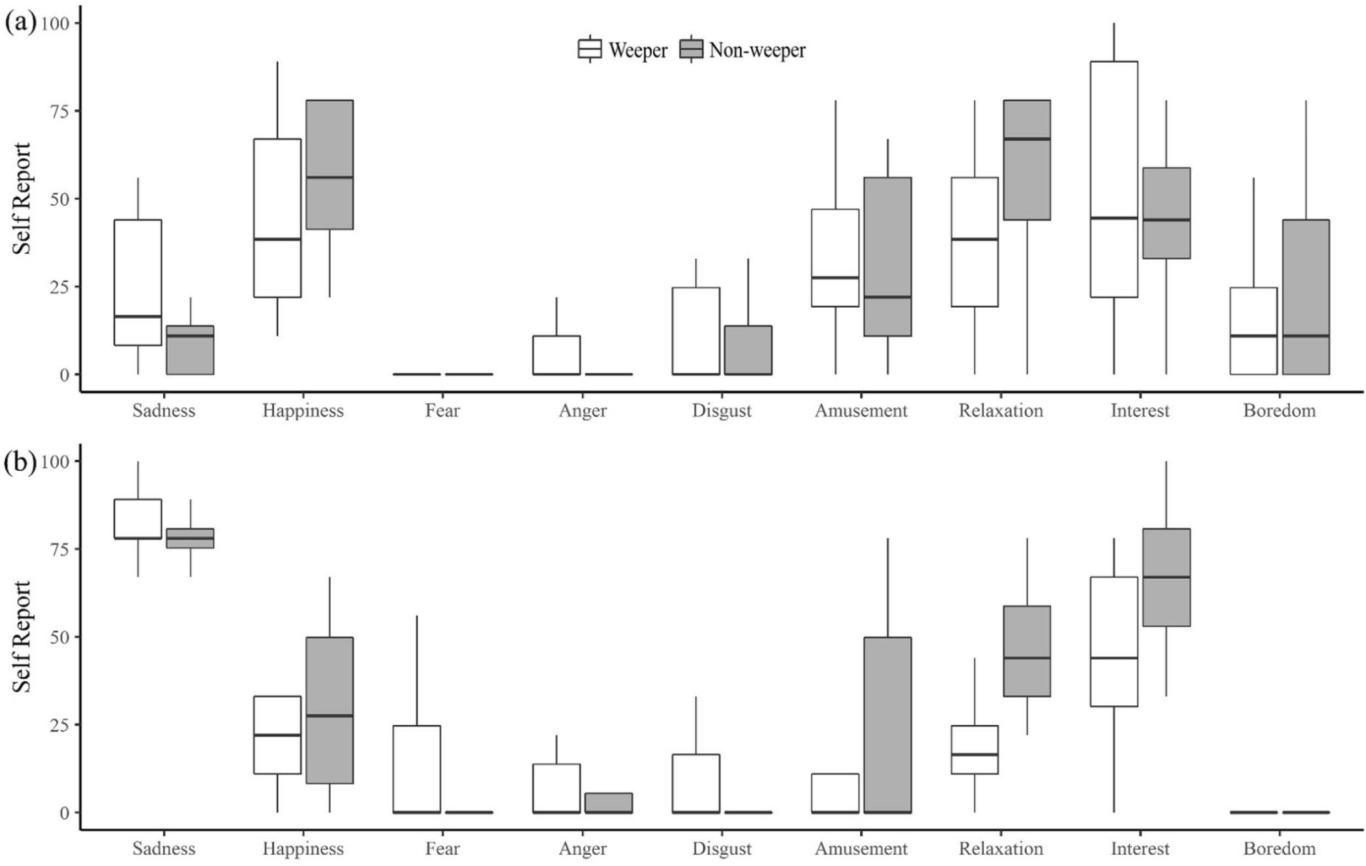
Self-Report Ratings for Nine Emotions in Response to a (**a**) Neutral Film and (**b**) Self-selected Sad Film for Weepers and Non-Weepers

Encoders reported moderately lower levels of happiness among non-weepers after watching the sad film compared to the neutral film (sadness est. = -24.17, *SE* = 8.59, *p* = .01), whilst there were large increases in self-reported sadness (sadness est. = 57.50, *SE* = 7.29, *p* < .001), and moderate increases in anger (sad film est. = 11.59, *SE* = 6.20, *p* = .06) and interest (sadness est. = 23.33, *SE* = 10.45, *p* = .03). Weepers were moderately less relaxed in the neutral condition than non-weepers (weeper est. = 18.50, *SE* = 8.42, *p* = .03), and reported a large decrease in interest during the sad film (sadness * weeper est. = -30.75, *SE* = 14.78, *p* = .04). Except for anger and fear ratings, the specific type of sad film contributed no random effects variance to the model, suggesting that the self-selected film-clips induced overall similar levels of emotion (see Fig. 3).

As the model reported in Table 2 only reports the specific effect of the sad film on non-weepers, an additional model was built for each of the subjective judgments to estimate the specific effect of the sad film on weepers. This model used the same fixed and random effects as above. Given that weepers acted as the reference group in this model, the intercept represents the self-report rating of weepers in the neutral condition. Weepers reported a significant increase in sadness (sadness est. = 58.67, *SE* = 6.49, *p* < .001), anger (sadness est. = 8.33, *SE* = 7.50, *p* = .019), and fear (sadness est. = 12.92, *SE* = 4.64, *p* = .004). Weepers also experienced lower levels of happiness (sad film est. = -24.17, *SE* = 7.50, *p* = .001), amusement (sadness est. = -27.75, *SE* = 8.19, *p* = .001), relaxation (sadness est. = -18.67, *SE* = 7.83, *p* = .017), and boredom (sadness est. = -21.25, *SE* = 9.09, *p* = .001) in the sad compared to the neutral condition. No significant effects emerged for weepers in terms of disgust (sadness est. = -5.5, *SE* = 8.08, *p* = .496) and interest (sadness est. = -7.42, *SE* = 9.16, *p* = .418).

### Stimulus ratings: valence, arousal, and genuineness

To analyze the human raters’ scores of positive and negative valence, arousal, and genuineness, a GLMM^3^ was built for each individual measure. We included a random intercept at the rater level to control for the effects of rater identity. Fixed effects were included for time phase (1: *neutral*, 2: *pre-sadness*, 3: *sadness*), whether the encoder was a weeper or non-weeper (tearing), and the interaction between time phase and tearing. For time phase, the neutral baseline episode (*neutral*) acted as a reference group, whilst non-weepers acted as a reference group for weepers. The intercept represents the mean rating for non-weepers during the neutral phase.

For negative valence, we observed a considerable increase across the time phases (pre-sadness est. = 4.92, *SE* = 1.80, *p* = .01, sadness est. = 6.48, *SE* = 1.80, *p* < .001), which was larger for weepers compared to non-weepers in both the pre-sadness phase (pre-sadness * weeper est. = 16.78, *SE* = 2.56, *p* < .001) and the sadness phase (sadness * weeper est. = 24.78, *SE* = 2.53, *p* < .001). There was no significant difference in negative valence between the pre-sadness and sadness phase for weepers (sadness * weeper est. = 5.48, *SE* = 2.97, *p* = .06^4^). While weepers were perceived similarly to non-weepers in the neutral baseline phase (weeper est. = -3.74, *SE* = 1.78, *p* = .04), they were rated increasingly more negative in the subsequent time phases. Attributions of positive valence were opposite to those of negative valence, with a reduction across the time phases (pre-sadness est. = -5.83, *SE* = 1.40, *p* < .001; sadness est. = -7.91, *SE* = 1.40, *p* < .001). This effect was significantly larger for weepers than non-weepers in the sadness phase (sadness * weeper est. = -6.16, *SE* = 1.98, *p* < .001), suggesting that human raters saw less positive affect in weepers but only after they had started weeping. In general, differences between weepers and non-weepers were larger in each time phase for attributions of negative (vs. positive) valence.

**Fig. 4:**
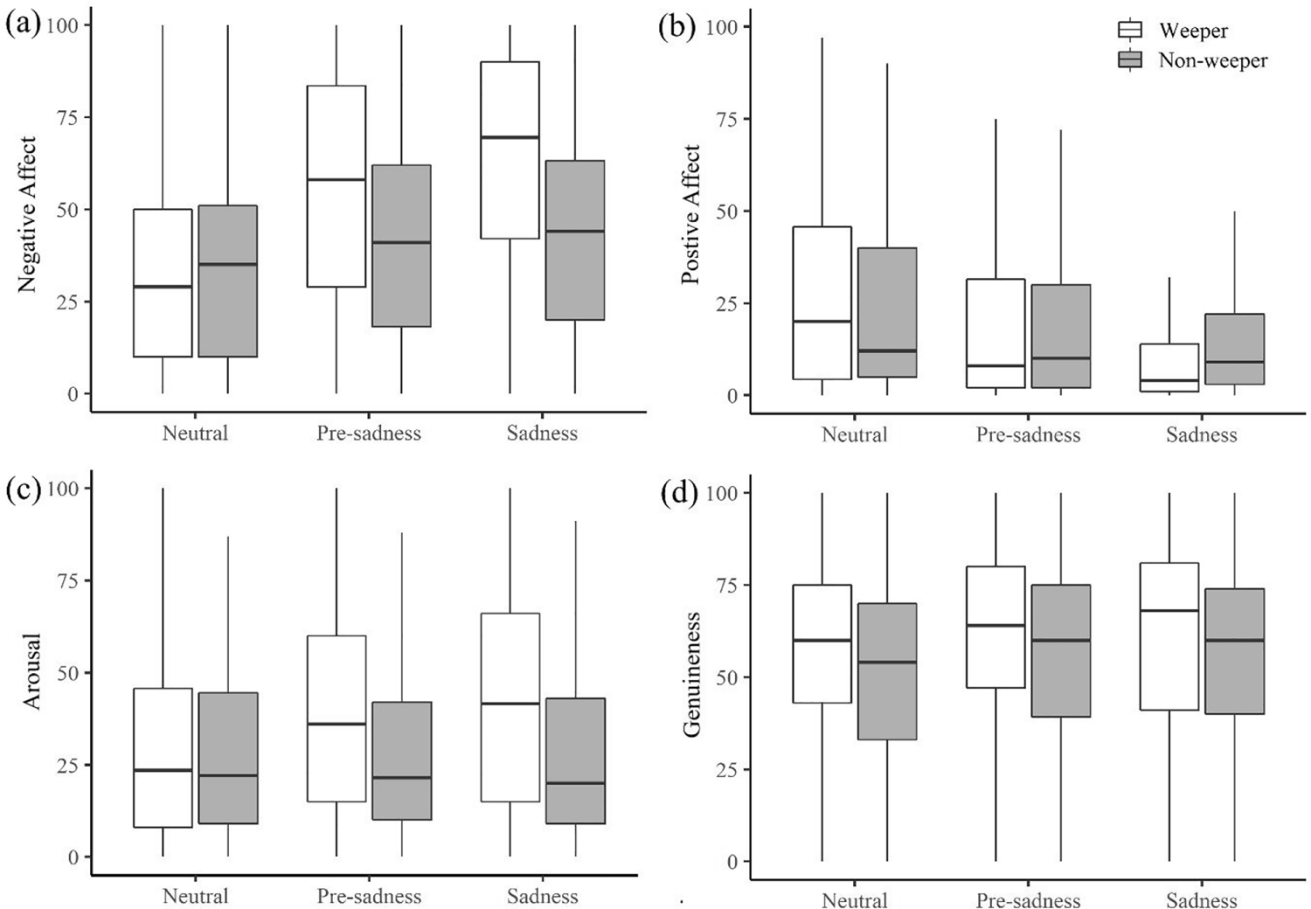
Observer Ratings of (**a**) Positive Valence, (b) Negative Valence, (del ) Arousal and (**d**) Genuineness at Neutral Baseline (phase 1), Pre-sadness (phase 2), and Sadness (phase 3) for Weepers and Non-Weepers

Arousal ratings were significantly higher for weepers (vs. non-weepers) in the pre-sadness phase (pre-sadness * weeper est. = 10.29, *SE* = 2.33, *p* < .001) and the sadness phase (sadness * weeper est. = 14.85, *SE* = 2.30, *p* < .001), although both time phases did not differ significantly from each other (sadness * weeper est. = 4.82, *SE* = 2.67, *p* = .07). Interestingly, arousal attributions for non-weeping encoders remained stable across the different time phases. Weepers were perceived as slightly more genuine than non-weepers (weeper est. = 3.58, *SE* = 1.59, *p* = .03); however, there was no indication that this was related to the act of weeping. Figure 4 shows the distribution of the ratings for weepers and non-weepers as a function of time phase^5^. Overall, judgments of valence and arousal were highly consistent, with clear differences between weepers and non-weepers over time. By contrast, the effects observed for emotion genuineness appeared to be more subtle (cf. Table 3 for complete GLMM results) Table 4.

**Table 3:** Estimates of Effects for Observer Ratings of Valence, Arousal, and Genuineness

Effects	Negative Valence			Positive Valence			Arousal			Genuineness		
	Est.	*SE*	*p*	Est.	*SE*	*p*	Est.	*SE*	*p*	Est.	*SE*	*p*
Fixed effects												
Intercept (β_0j_)	36.56	1.99		23.19	1.43		28.06	1.82		53.51	1.85	
Pre-sadness	**4.92**	1.80	**.01**	**-5.83**	1.40	**<.001**	1.02	1.64	.53	2.85	1.61	.08
Sadness	**6.48**	1.80	**<.001**	**-7.91**	1.40	**<.001**	-0.70	1.64	.67	3.06	1.61	.06
Weeper	**-3.74**	1.78	**.04**	**3.32**	1.39	**.02**	0.36	1.62	.82	**3.58**	1.59	**.03**
Pre-sadness * Weeper	**16.78**	2.56	**<.001**	-1.53	2.00	.44	**10.29**	2.33	**<.001**	1.19	2.29	.60
Sadness * Weeper	**24.78**	2.53	**<.001**	**-6.16**	1.98	**<.001**	**14.85**	2.30	**<.001**	0.98	2.26	.67
Random Effect												
Random Intercept (*U*_*0j*_*)*	214.50	35.08		98.49	16.69		182.13	29.85		193.43	31.33	
Residual (***e***_ij_)	561.64	17.39		343.16	10.62		466.05	14.41		449.54	13.91	
-2*loglikelihood	20226.36			19110.35			19829.97			19750.19		

Estimates printed in bold indicate statistically significant effects at *p* < .05, with the neutral phase and non-weepers acting as reference groups. *Pre-sadness* represents the effect of the pre-sad phase for non-weepers compared to the neutral phase. *Sadness* represents the effect of the sad phase compared to the neutral phase for non-weepers. *Weeper* represents the effect of being a weeper during the neutral phase. *Pre-sadness* * *Weeper* represents the effect of weepers compared to non-weepers in the pre-sadness phase. *Sadness* * *Weeper* represents the effect of weepers compared to non-weepers in the sadness phase.

**Table 4:** Estimates of Effects for Observer Ratings of Discrete Emotions

Effects	Neutral			Sadness			Happiness			Fear			Anger			Disgust			Surprise		
	Est.	*SE*	*p*	Est.	*SE*	*p*	Est.	*SE*	*p*	Est.	*SE*	*p*	Est.	*SE*	*p*	Est.	*SE*	*p*	Est.	*SE*	*p*
Fixed effects																					
Intercept (β_0j_)	53.56	2.00		16.14	1.82		10.98	1.02		3.70	0.81		5.09	0.74		4.39	0.66		6.22	0.66	
Pre-sadness	**-8.51**	2.35	**<.001**	**9.12**	1.97	**<.001**	**-5.06**	1.31	**<.001**	**4.47**	1.03	**<.001**	0.54	0.89	.55	**1.69**	0.84	**.04**	**-2.36**	0.84	**.01**
Sadness	**-7.57**	2.35	**<.001**	**12.00**	1.97	**<.001**	**-6.99**	1.31	**<.001**	**3.38**	1.03	**<.001**	-0.21	0.89	.82	1.56	0.84	.06	**-2.31**	0.84	**.01**
Weeper	2.92	2.33	.21	-1.34	1.95	.49	1.01	1.30	.44	-0.49	1.03	.63	**-2.01**	0.89	**.02**	-0.07	0.84	.94	0.09	0.84	.92
Pre-sadness * Weeper	**-29.22**	3.34	**<.001**	**23.50**	2.79	**<.001**	**4.76**	1.86	**.01**	0.94	1.47	.52	0.50	1.27	.70	-1.96	1.20	.10	1.29	1.19	.28
Sadness * Weeper	**-34.91**	3.31	**<.001**	**31.87**	2.77	**<.001**	2.42	1.85	.19	2.35	1.46	.11	1.36	1.26	.28	**-2.89**	1.19	**.02**	-0.35	1.19	.77
Random Effect																					
Random Intercept (*U*_*0j*_*)*	116.44	23.21		126.62	22.91		17.70	4.50		11.40	2.86		13.71	2.89		7.09	1.83		7.13	1.84	
Residual (***e***_ij_)	967.32	29.91		675.33	20.88		303.02	9.37		188.75	5.84		140.09	4.33		125.42	3.88		125.19	3.87	
-2*loglikelihood	21335.49			20582.23			18756.62			17724.55			17101.87			16828.22			21335.49		

Estimates printed in bold indicate statistically significant effects at *p* < .05, with the neutral phase and non-weepers acting as reference groups. *Pre-sadness* represents the effect of the pre-sad phase for non-weepers compared to the neutral phase. *Sadness* represents the effect of the sad phase compared to the neutral phase for non-weepers. *Weeper* represents the effect of being a weeper during the neutral phase. *Pre-sadness* * *Weeper* represents the effect of weepers compared to non-weepers in the pre-sadness phase. *Sadness* * *Weeper* represents the effect of weepers compared to non-weepers in the sadness phase.

### Stimulus ratings: discrete emotions

To analyse the human raters’ emotion scores, a GLMM was built for each of the seven discrete emotions. We included a random intercept at the rater level to control for the effects of rater identity. Fixed effects were included for time phase (1: *neutral*, 2: *pre-sadness*, 3: *sadness*), whether the encoder was a weeper or non-weeper (*tearing*), and the interaction between time phase and tearing. For time phase, the neutral baseline episode (*neutral*) acted as a reference group, whilst non-weepers acted as a reference group for weepers. The intercept represents the mean rating for non-weepers during the neutral phase.

**Fig. 5:**
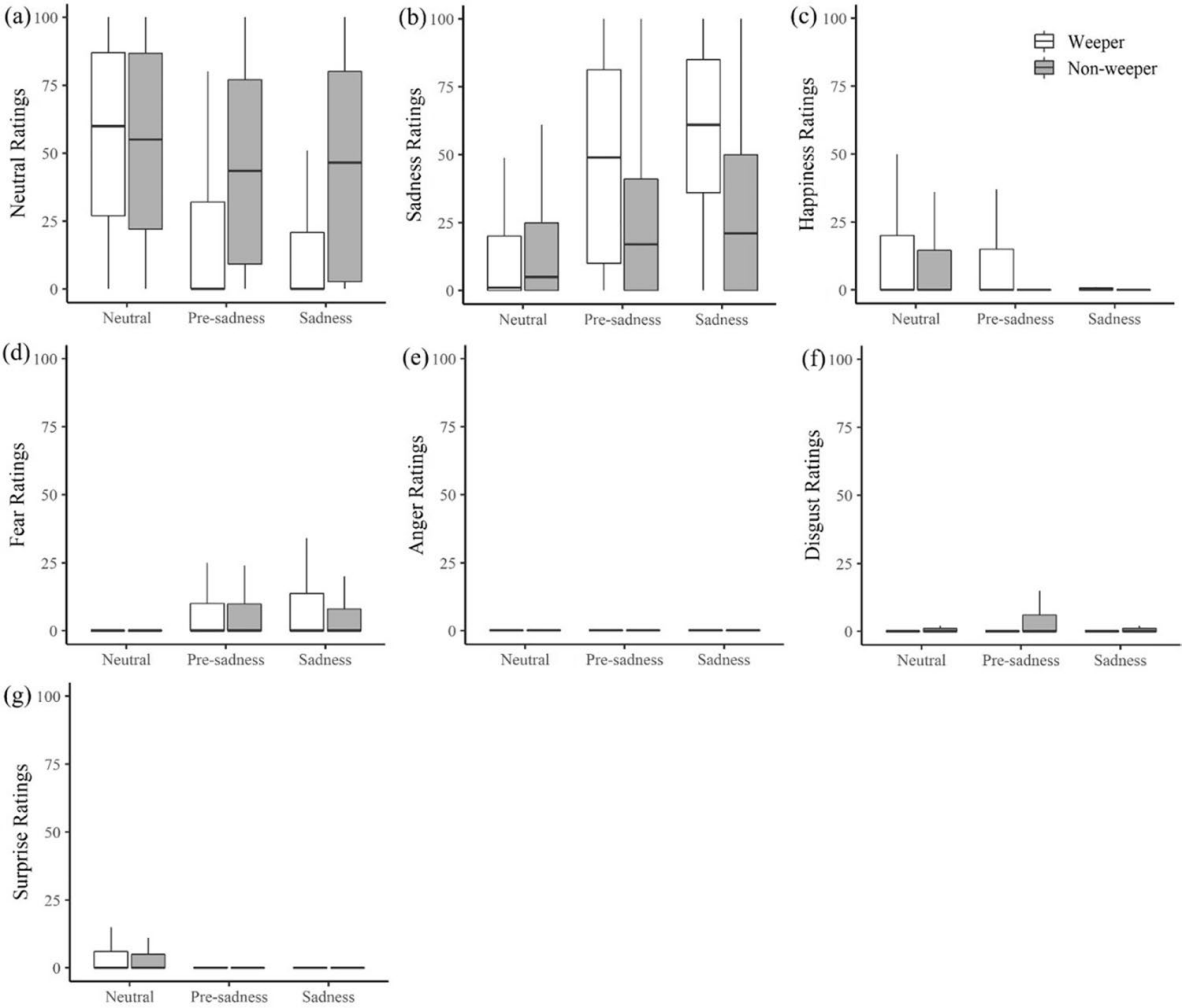
Observer Ratings for the Six Basic Emotions and Neutral Emotion at Each Time Phase for Weepers and Non-Weepers

For sadness ratings, there was a significant increase across the time phases (pre-sadness est. = 9.12, *SE* = 1.97, *p* < .001; sadness est. = 12.00, *SE* = 1.97, *p* < .001), which was substantially larger when the encoder wept (pre-sadness * weeper est. = 23.50, *SE* = 2.79, *p* < .001; sadness * weeper est. = 31.87, *SE* = 2.77, *p* < .001). Sadness ratings were also significantly higher in the sadness than pre-sadness phase for weepers (sadness * weeper est. = 7.36, *SE* = 2.97, *p* = .013). While there was no significant difference between weepers and non-weepers in the neutral baseline phase (weeper est. = -1.34, *SE* = 1.95, *p* = .49), more sadness was attributed as a function of time phase and tearing. Ratings of neutral emotion were opposite to those of sadness and decreased over time (pre-sadness est. = -8.51, *SE* = 2.35, *p* < .001; sadness est. = -7.57, *SE* = 2.35, *p* < .001), with a larger reduction if the encoder wept (pre-sadness * weeper est. = -29.22, *SE* = 3.34, *p* < .001; sadness * weeper est. = -34.91, *SE* = 3.31, *p* < .001). The pre-sadness and sadness phase did not differ significantly from each other in terms of neutral emotion (sadness * weeper est. = -4.58, *SE* = 3.46, *p* = .19). Weepers and non-weepers were perceived as similarly neutral during the neutral baseline phase (weeper est. = 2.92, *SE* = 2.33, *p* = .21). Figure 5 illustrates this antagonistic relation of sadness and neutral scores across the three time phases.

While there were clear effects for the remaining discrete emotion categories, they played a relatively minor role in terms of the size of the estimates. Happiness ratings decreased in both the pre-sadness (pre-sadness est. = -5.06, *SE =* 1.97, *p* < .001) and sadness (sadness est. = -6.99, *SE* = 1.31, *p* < .001) phases, with small increases in weepers’ happiness ratings in the pre-sadness phase (weeper*pre-sadness est. = 4.76, *SE* = 1.86, *p* =.01). Anger ratings were reduced for weepers in the neutral phase (weeper est. = -2.01, *SE* = 0.89, *p* =.02), and fear ratings increased during the sad film in both time phases (pre-sadness est. = 4.47, *SE =* 1.03, *p* < .001; sadness est. = 3.38, *SE* = 3.38, *p* < .001). There was a small increase in disgust ratings in the pre-sadness phase (pre-sadness est. = 1.69, *SE* = 0.84, *p* = .04), and a significant decrease in disgust ratings for weepers in the sadness phase (weeper*sadness est. = -2.89, *SE* = 1.19, *p* = .02). Finally, surprise ratings decreased during the sad film in both time phases (pre-sadness est. = -2.36, *SE* = 0.84, *p* = .01; sadness est. = -2.31, *SE* = 0.84, *p* = .01). It should be noted that the estimate sizes were very small and significant effects may have been the product of low ratings at the intercept (neutral baseline phase).

## Discussion

In this article, we introduced the PDSTD as a new resource for crying research. Up to now, most research was limited to the use of posed or digitally manipulated materials (Krivan & Thomas, 2020). Moreover, stimulus sets were dominated by static images (Küster, 2015), which lack information about the behavioral antecedents of crying. While such an approach was sufficient to demonstrate the *tear effect* (Cornelius et al., 2000; Provine et al., 2009), comparable studies using dynamic and spontaneous stimuli have been missing so far. The present work reports the first validated and openly accessible database containing spontaneously elicited tears and dynamic sadness expressions. For establishing norming data, we adopted a two-fold validation procedure involving the original encoders as well as naïve observers. Furthermore, a multilevel modelling approach was employed to account for random variance from participants and target category.

With regard to self-reports of emotional experience, results showed that the sadness manipulation was successful. Encoders reported high levels of sadness, with a reduction in happiness, after watching the self-selected sad film-clip. Ratings significantly differed from those in response to the neutral (control) clip for both weepers and non-weepers. While weepers felt less interested during the sad film than non-weepers, this effect appeared to be driven mainly by the increase of interest from non-weepers. Also, non-weepers felt generally more relaxed than weepers. It is possible that the greater interest of non-weepers in the sad film reflects a different, less aroused, focus of attention when processing the stimulus content. However, we did not obtain any further data in this study that would allow us to test such assumptions. Clearly, more research is needed in the future to explore the psychological mechanisms underlying tear production. Clearly, more research is needed in the future to explore the psychological mechanisms underlying tear production. The lack of substantial differences between weepers and non-weepers in self-reports during the sad phase largely speaks against the notion of a cathartic function of weeping for intrapersonal emotion regulation (Breuer & Freud, 1895/2009; Vingerhoets, 2013).

Observer ratings by naïve judges revealed a similar pattern of results as those obtained for the self-reports. Specifically, the sad film-clip led to substantial increases in perceived sadness and negative valence compared to the neutral film. Furthermore, ratings of positive valence, happiness and neutral emotion were significantly reduced, indicating that the sadness manipulation was effective in driving differences in perception.

Notably, we found significant differences between weepers and non-weepers as a function of time phase. While the two target groups were comparable during the neutral phase, weepers received higher ratings of sadness, negative valence, arousal, and lower ratings of neutral emotion in the pre-sadness and sadness phase. In addition, positive valence ratings decreased, and sadness ratings increased for weepers in the sadness phase, i.e., after encoders had started to weep. Together these findings provide evidence in support of the *tear effect* (Cornelius et al., 2000; Provine et al., 2009), suggesting that the shedding of tears by weepers enhances sadness perceptions. To our knowledge, this is the first study demonstrating the effect in response to spontaneous and dynamic stimuli.

Using dynamic expressions, we could further explore whether observers were sensitive to the behavioral antecedents of crying (Bylsma et al., 2021; Vingerhoets, 2013; Vingerhoets & Bylsma, 2016). As expected, substantial differences in emotion attribution occurred between weepers and non-weepers already during the pre-sadness phase. Spotting episodes of “near-weeping” could be highly relevant for social interaction partners who may want to intervene before a highly intense experience crosses a certain threshold. The temporal context of the pre-sadness phase might reveal important cues that have predictive value for the occurrence of tears. Although effects were observed for several measures in the present study, the overall pattern of results was dominated by perceived sadness. This points towards the unique value of tears in the context of facial expressions (Ito et al., 2019; Reed et al., 2015). In accordance with the sadness enhancing hypothesis by Gračanin et al. (2021), the signaling function of tears might thus be specifically tied to the perception of sadness.

Future research might be aimed at exploring the distinct behavioral cues that impact observer responses. While the present research made use of infrared thermal imaging to determine the presence of tears, the interplay of weeping, facial expressions, and other nonverbal behaviors (i.e., face touching, Znoj, 1997) is likely to be of importance. For this, a fine-grained behavioral analysis using manual or automated coding systems (e.g., FACS, Dupré et al., 2020; Ekman et al., 2002; Krumhuber et al., 2021) is needed to access the relative contribution of each cue to observer ratings. Future work might aim to record spontaneous crying “in the wild”, e.g., during therapy, funerals, or in everyday contexts that frequently elicit crying. While this could pose substantial ethical challenges, if successful, such an approach might further improve the ecological validity of crying research compared to the present laboratory setting.

While weeping is difficult to elicit in the laboratory (Gračanin et al., 2015), the self-selected sad film-clips achieved this in half of the participants included in the database. It must be noted that all our participants were White and female; hence our stimulus set is not diverse in terms of gender and race. Although participants knew that the research was about crying, sadness and responses to sad films, weeping was not necessary or in any way required. Nonetheless, we cannot entirely rule out the possibility of self-selection biases. Prior work suggests that only a small percentage of male participants would cry in this type of setting (Gračanin et al., 2015), which has resulted in most laboratory studies focusing on an exclusively female population (e.g., Sharman et al., 2019). Our exploratory results for perceived genuineness revealed comparatively high ratings, supporting our aim to provide a well-controlled but spontaneous database. Having such well-normed and ecologically valid stimuli will aid future researchers using more sophisticated methods, further advancing our knowledge of how tears function as socio-emotional signals in sadness expressions. Towards this aim, our database should be compared to other new datasets, which could feature broader or different populations (e.g., male, non-White, or elderly people). Likewise, future research may collect additional rating data on the PDSTD from more diverse target groups to study how crying perceptions may generalize or change across the lifespan or between cultures. This may allow researchers to address many of the long-standing questions concerning individual and group differences in crying (Vingerhoets, 2013).

## Footnotes

1 The participants whose videos are included in this database signed an informed consent form as well as an extended consent covering the use of their videos for creating the database as well as the following purposes (for further details see https://osf.io/3r8d9/): 1. The videos may be used in future experiments where they will be seen by others 2. Anonymized results of studies may be published and / or presented at meetings or academic conferences and may be provided to research commissioners or funders. 3. Participants agreed to their data being retained for any future research that has been approved by a Research Ethics Committee. 4. They consented for videos of them to be taken during the experiment for use in scientific presentations and publications.

2 As GLMMs do not compute traditional main effects and interaction effects, we performed additional ANOVAs for all analyses (see supplementary materials, table S2, https://osf.io/uyjeg/?view_only=24474ec8d75949ccb9a8243651db0abf). Overall, these results resemble the present GLMM models, except that they cannot account for any of the 2nd level sources of variance discussed above.

3 The random intercept at the level of rater allowed us to estimate the variance due to each rater. Consistent with assumptions of the use of GLMM there was a substantial amount of rater-level variance for each of the four measures: negative valence = 29.6%, positive valence = 22.3%, arousal = 28.1%, genuineness = 30.1%).

4 All estimates and p-values that are reported in the text, but not included in tables 3 or 4, were computed by modifying the respective models to use the pre-sadness phase as the reference category.

5 A further breakdown by individual encoder is provided in the Supplementary Materials (table S1, https://osf.io/uyjeg/?view_only=24474ec8d75949ccb9a8243651db0abf).
